# A favorable suture method for size-mismatched nerve transfer: comparison with standard perineural suture in an experimental rat study

**DOI:** 10.1186/s13018-023-04123-7

**Published:** 2023-09-06

**Authors:** Sho Iwabuchi, Yuki Hara, Yuichi Yoshii, Akira Ikumi, Hajime Mishima, Masashi Yamazaki

**Affiliations:** 1 Department of Orthopaedic Surgery, Faculty of Medicine, University of Tsukuba, Tsukuba, Ibaraki Japan; 2 Department of Orthopaedic Surgery, National Center of Neurology and Psychiatry, Kodaira, Tokyo Japan; 3 Department of Orthopaedic Surgery, Tokyo Medical University Ibaraki Medical Center, 3-20-1 Chuo, Ami, Inashiki, Ibaraki 300-0395 Japan

**Keywords:** Brachial plexus injury, Peripheral nerve injury, Nerve transfer, Suture method, Nerve regeneration, Size-mismatched nerve suture

## Abstract

**Background:**

In nerve transfer for peripheral nerve injury, it is sometimes necessary to suture size-mismatched nerves. In 1993, a favorable suture method called the Ochiai suture method for size-mismatched nerve transfer was reported. However, there is currently a lack of substantial evidence beyond the original report. Therefore, this study aimed to verify the advantages of using the Ochiai suture method for size-mismatched nerve transfer.

**Methods:**

A total of 18 rats were evaluated in this study and randomly divided into two groups. All rats underwent femoral to sciatic nerve transfer. Specifically, group A (*n* = 10) underwent the Ochiai suture method, while group B (*n* = 8) underwent the perineural suture method. After 12 weeks postoperatively, we conducted the sciatic functional index (SFI) test, measured muscle wet-weight, and performed histological evaluations. All data were compared between the two groups, with Welch’s t test for normally distributed data and Mann-Whitney's U test for non-normally distributed data. Statistical significance was set at *p* < 0.05.

**Results:**

The mean number of axons was significantly greater in group A than in group B at 5 mm distal to the stump (*p* = 0.04). Additionally, the average axonal diameter was significantly greater in group A than in group B at 5 mm and 10 mm distal to the stump (*p* < 0.01 and *p* < 0.01, respectively). However, the SFI test and measured muscle wet-weight values showed no significant differences between the two groups.

**Conclusions:**

Our study revealed that the Ochiai suture method for size-mismatched nerve transfer in rats increases the regenerative axon numbers and diameters. These findings suggest that the Ochiai suture method could be a valuable approach for achieving effective motor function restoration in cases of size-mismatched nerve transfer.

## Background

Nerve transfer is a valuable treatment option for peripheral nerve injury, especially in cases of root avulsion in brachial plexus injury. Intercostal-to-musculocutaneous nerve transfer was first reported in 1963 [1]. Since then, several combinations of donor and recipient nerves have been reported [2–6].

In nerve transfer involving the suturing of two different nerves, large differences in the diameter of the nerves can occur, especially in intercostal-to-musculocutaneous nerve transfer. As a result, if the standard perineural nerve suture is performed, ideal nerve regeneration may not occur because the regenerative axon from the smaller donor nerve can only incline toward a small area of the larger recipient nerve. Despite these challenges, details of a suitable suture method have not been described in most studies on intercostal-to-musculocutaneous transfer.

To address this problem, Ochiai introduced a unique nerve suture method for size-mismatched nerve transfer [7], which can be used to suture different diameter nerves without deviation. In his study, 91% (10 of 11) patients who underwent intercostal-to-musculocutaneous nerve transfer achieved more than M3 elbow flexion power. Similarly, in a recent systematic review, 77.7% (539 of 703) of patients who underwent intercostal-to-musculocutaneous achieved more than M3 elbow flexion power [8]. When compared to this systematic review, Ochiai’s report showed excellent results.

Although the specific advantages of Ochiai’s method remain to be thoroughly validated, and there is limited evidence beyond the original report regarding this technique's efficacy, we hypothesized that Ochiai’s method would lead to better axonal regeneration and provide better results than any other method.

Therefore, this study aimed to evaluate this method using nerve transfer in rat models and verify the advantages of using the Ochiai suture method for size-mismatched nerve transfer.

## Methods

### Animal model

Twenty 8-week-old Wistar rats were used in this experimental study (weight: 210–260 g, CLEA Japan, Inc.). They were randomly divided into two groups (group A: Ochiai suture method, and group B: perineural suture method). All rats were kept in a smooth-floor cage under a 14-h light/10-h dark cycle.

Ethical considerations were strictly followed, and animals were humanely treated after receiving approval from the Institutional Animal Care and Use Committee of our institute. The experiments were conducted in accordance with the Regulation for Animal Experiments of our university and the Fundamental Guidelines for Proper Conduct of Animal Experiment and Related Activities in Academic Research Institutions under the jurisdiction of the Ministry of Education, Culture, Sports, Science, and Technology.

### Experimental surgery

**Fig. 1:**
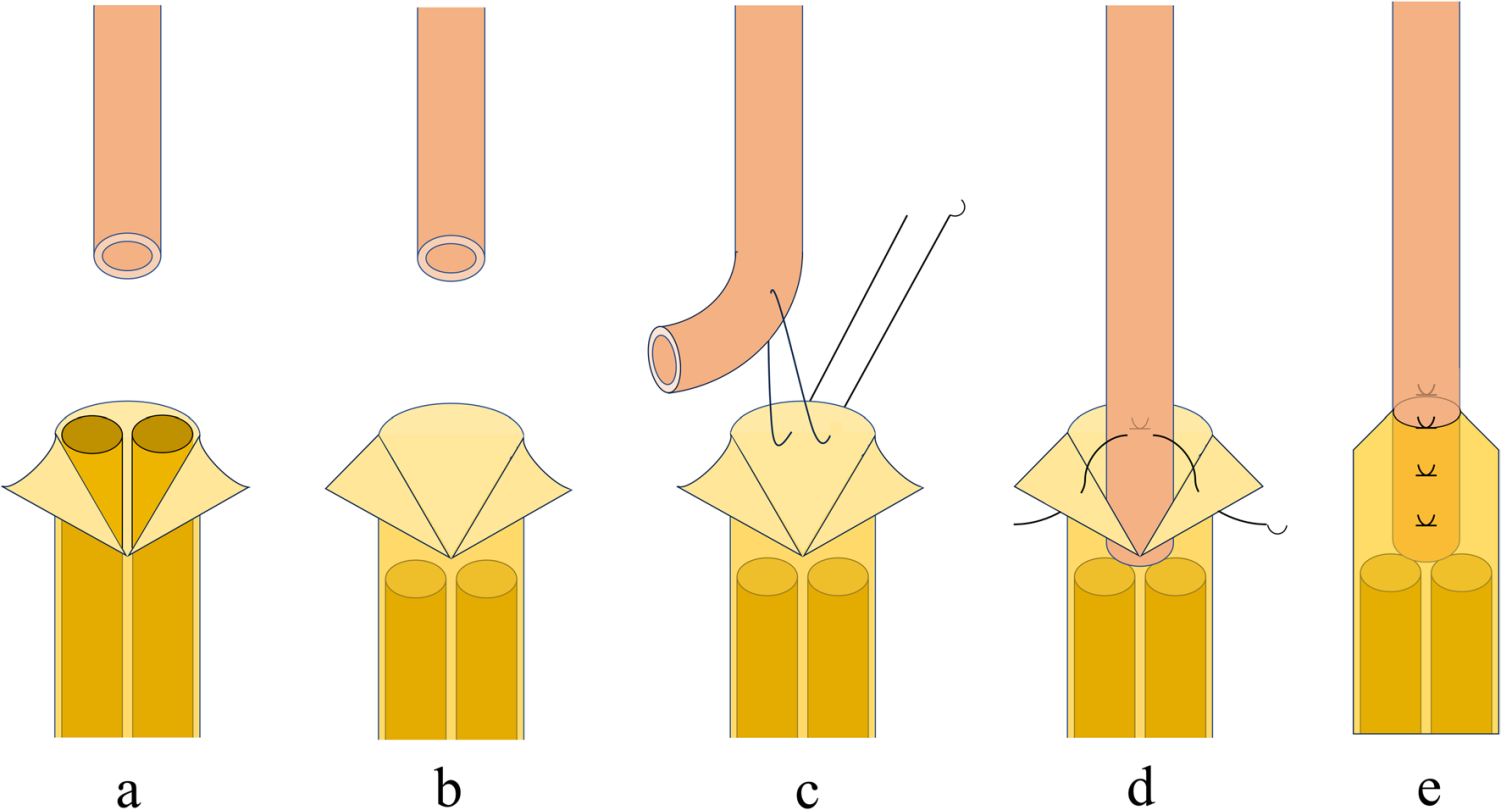
A procedure using the Ochiai suture method. **a**: The femoral nerve (up) and the sciatic nerve (down). An epineurium of the sciatic nerve is cut and spread out in a skirt-like fashion. **b**: Ends of the funiculus are cut to draw it back into the epineurium. **c**: The sides of the femoral nerve bundle are sutured to the epineural skirt center. **d**: The opposite side of the suture in the procedure was sutured to the corner of the epineural skirt. **e**: A slit of the epineurium is closed by suture and covered with a femoral nerve bundle

**Fig. 2:**
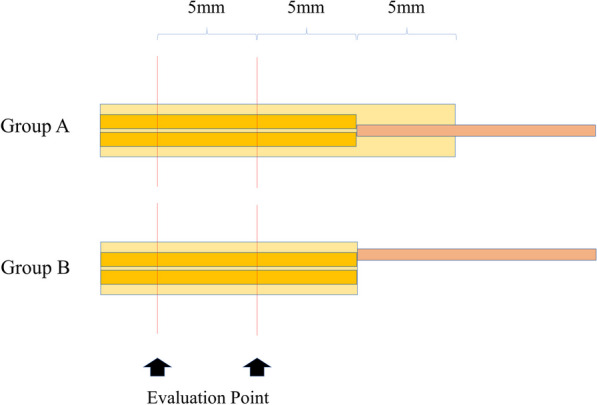
The sciatic nerve (left) and the femoral nerve (right). The evaluation points are 5 mm and 10 mm distal from the suture points. All points were at the same level in each group

**Fig. 3:**
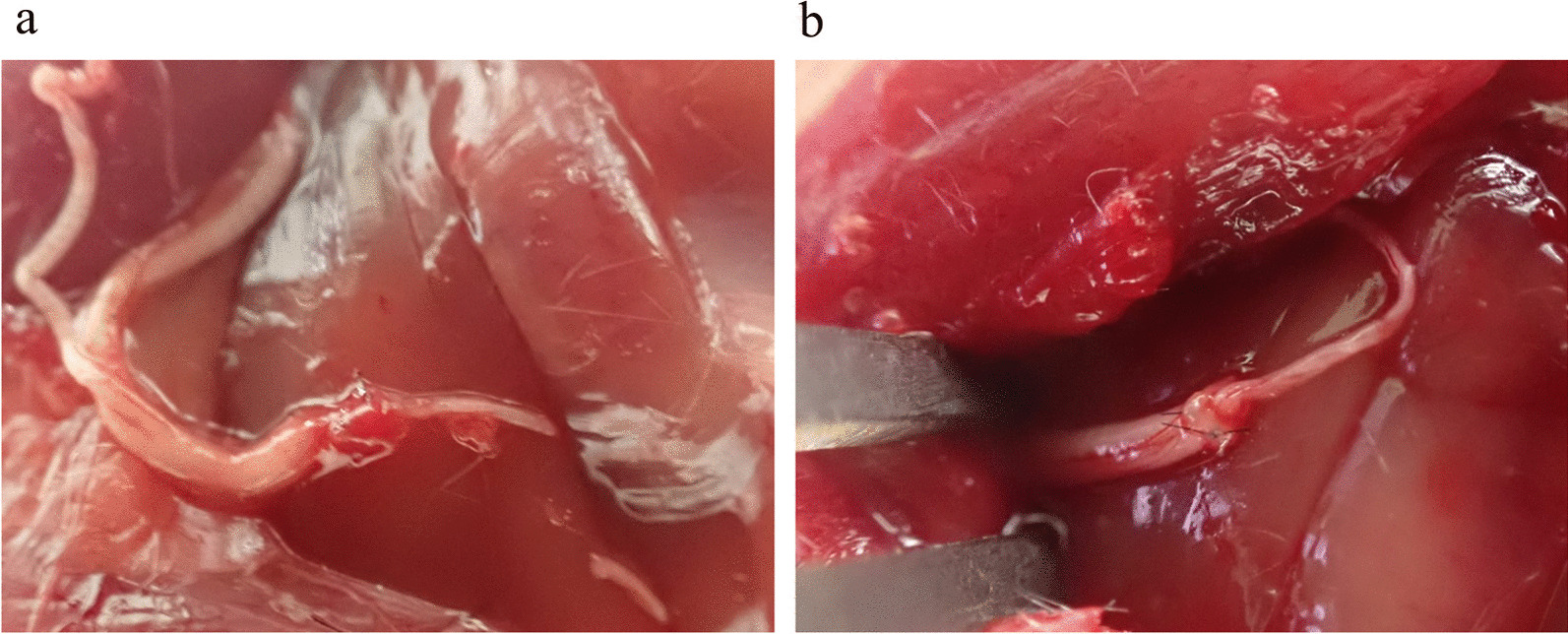
Intraoperative findings. **a**: Group A (Ochiai suture method). **b**: Group B (Epineural suture method)

The rats received general anesthesia through an intraperitoneal injection of ketamine hydrochloride (50 mg/kg; Ketalar, Daiichi Sankyo Propharma Co., Ltd., Japan) and xylazine hydrochloride (0.12 mg/kg; Selactar, Bayer Yakuhin, Ltd., Japan). The left sciatic nerve was exposed via an incision made from the lateral femur to the tibia and then divided between the quadriceps and biceps muscles. The sciatic nerve was resected at the point of the lateral rotator group of the hip, and the femoral nerve was then exposed as distally as possible and resected. The resecting point was set at the same level in all rats. The proximal end of the femoral nerve was then moved to the distal end of the sciatic nerve through the biceps femoris muscles. The connection between the distal end of the sciatic nerve and the proximal end of the femoral nerve was sutured using two different methods. In group A, the Ochiai suture method was employed. A slit in the epineurium was made 5 mm from the end of the recipient sciatic nerve and spread out in a skirt-like fashion (Fig. 1a). The funicular ends of the nerve were cut to draw it back into epineurium (Fig. 1b). Notably, one side of the sciatic nerve was sutured to the center of the epineural skirt at 5 mm proximal from the stump (Fig. 1c), while the opposite side of the nerve was sutured to the corner of the epineural skirt (Fig. 1d). Afterward, the epineural slit was closed with two sutures and covered with a sciatic nerve (Fig. 1e). In group B, to suture at the same level as in group A, 5 mm from the end of the sciatic nerve was resected, and a perineural suture was performed (Figs. 2 and 3). All sutures were made using Nylon 9–0. After 12 weeks postoperatively, the following evaluation was performed to assess the outcomes:

- Sciatic functional index (SFI) test

To evaluate the gait and functional recovery of the rats, the planta pedis of each rat were painted with black ink, and they were allowed to walk in a straight line on a piece of paper. Clear footprints were selected, and three different parameters were measured [9], including (1) distance from the heel to the third toe (print length; PL); (2) distance from the first to the fifth toe (toe spread; TS); and (3) distance from the second to the fourth toe (intermediate toe spread; ITS). After that, the SFI was calculated using the following formula: SFI = − 38.3 ((EPL-NPL)/NPL) + 109.5 ((ETS-NTS)/NTS) + 13.3 ((EITS-NITS)/NITS) − 8.8 (where E is the experimental side, and N is the normal side).

(b) Muscle wet-weight measurements

The whole gastrocnemius muscle was harvested and weighed on an electronic precision scale (AB204; Mettler-Toledo International Inc., Switzerland). Data recorded were evaluated as a percentage of the contralateral muscle weight.

(c) Histological evaluation

The sciatic nerve was harvested from the proximal stump 5 mm and 10 mm distally. The harvested nerve was then fixed immediately with glutaraldehyde solution 2% in 0.1 M Phosphate Buffer Solution (PBS) and left overnight at 4 °C. Following this, the specimens were washed with PBS thrice for 15 min each, fixed for 2 h in 2% tetroxide osmium, and washed with PBS twice for 15 min each. They were then dehydrated using increasing concentrations of ethanol and embedded in Epon 812 (TABB Aldermaston, United Kingdom). Afterward, 1 μm transactional slices were made at 5 mm and 10 mm distal to the sutured point, stained with toluidine blue, and examined under a light microscope (BX-51, Olympus, Japan). The total number of myelinated axons and axonal diameter were measured at 400 × magnification and analyzed in five random fields. The number of myelinated axons was calculated per unit area (/mm^2^), and the average axonal diameter was measured (μm).

### Statistical analysis

Data are expressed as means (standard deviation). To assess the normality of the datasets, the Shapiro–Wilk test was used. Welch’s t test (or Mann–Whitney U tests if the data were not normally distributed) was used for comparisons between groups A and B. Statistical significance was set at *p* < 0.05. All statistical analyses were performed using IBM SPSS Statistics 27.0.

## Results

In group B, two rats were excluded from the study, one due to a wound infection secondary to auto-mutilation and another due to a ruptured nerve during observation. Ultimately, 18 rats were included in this study (group A = 10 and group B = 8).

**Fig. 4:**
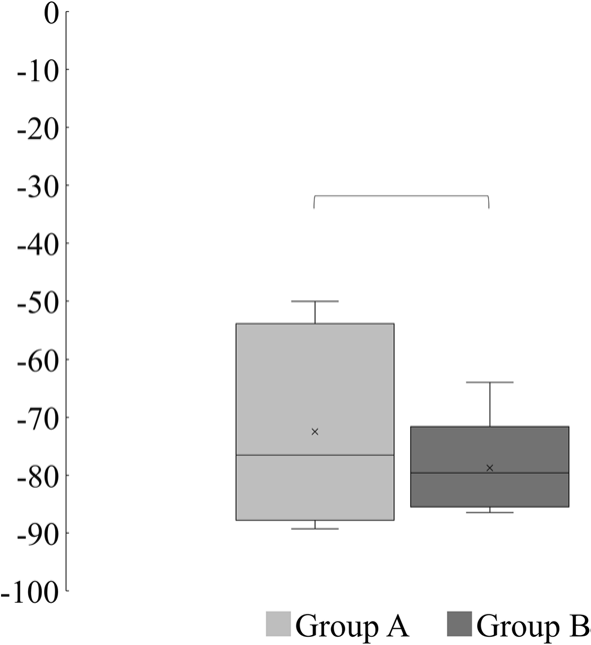
Comparison of SFI. There was no significant difference between the two groups. ^*^Welch’s t test

The SFI values were − 75.49 ± 14.95 in group A and − 78.75 ± 8.84 in group B, and no significant difference was observed between the two groups (*p* = 0.63) (Fig. 4).

**Fig. 5:**
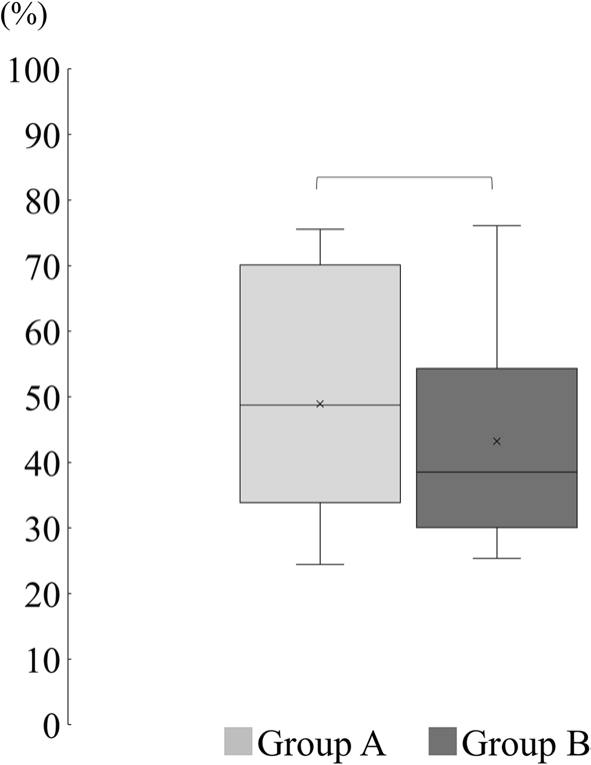
Comparison of the mean muscle wet-weight value on the contralateral side. There was no significant difference between the two groups. ^*^Welch’s t test

The mean muscle wet-weight on the contralateral side was 48.93 ± 18.39% in group A and 43.23 ± 16.61% in group B, and no significant difference was observed between the two groups (*p* = 0.5) (Fig. 5).

**Fig. 6:**
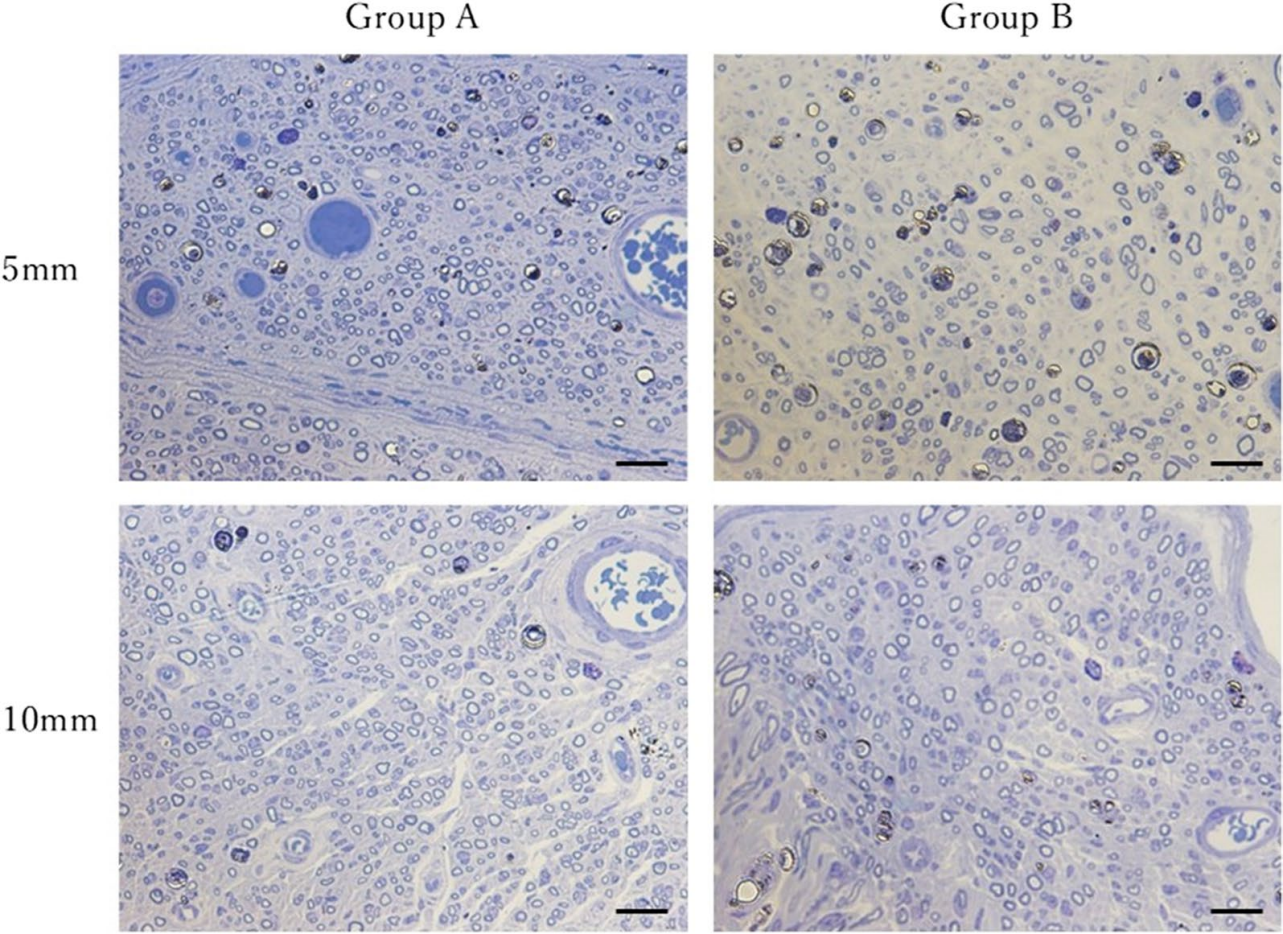
Histological appearance (toluidine blue, 400 × , scale bar = 20 µm)

**Fig. 7:**
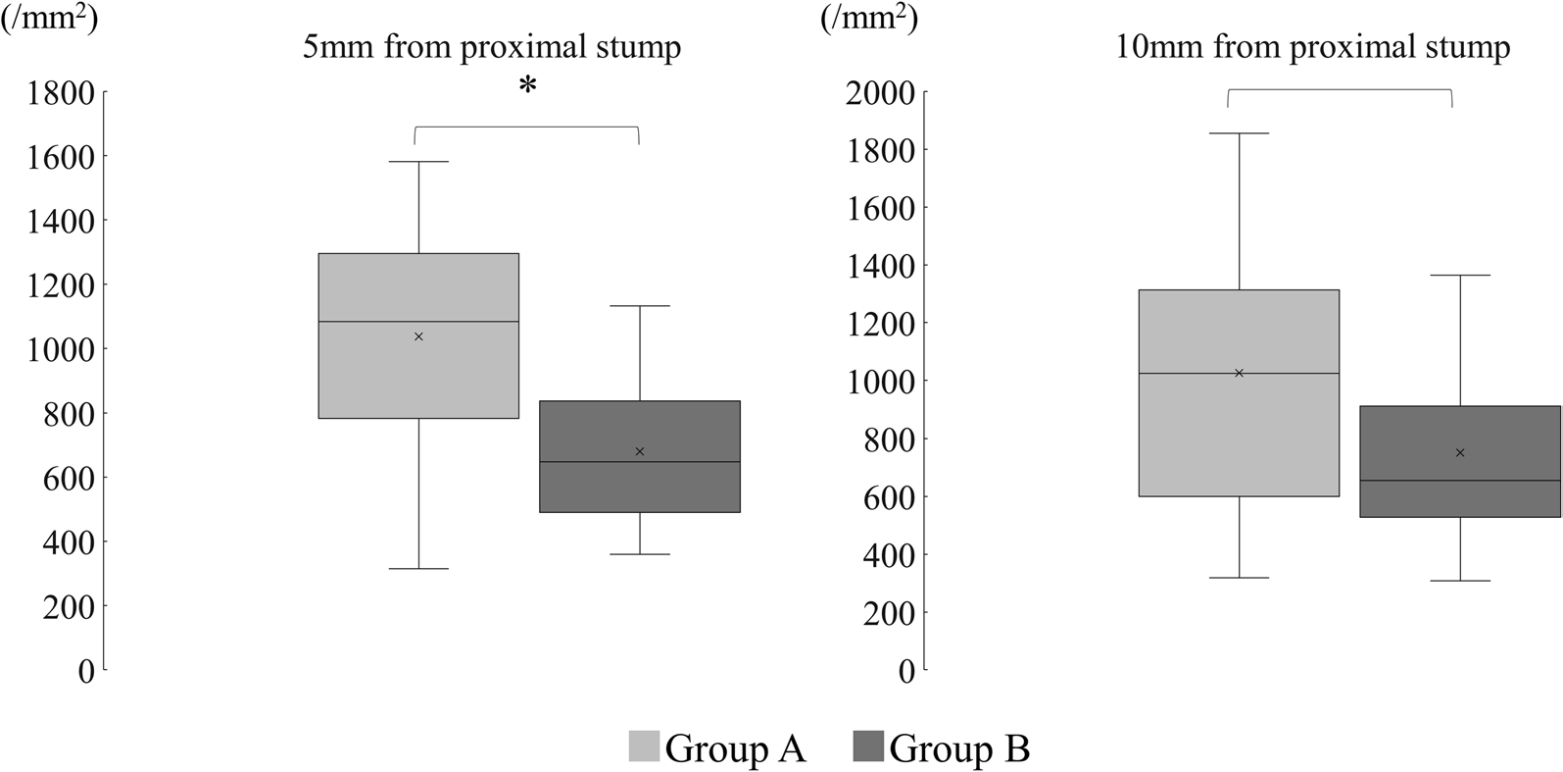
Comparison of the axonal number. Significant differences existed between the two groups at 5 mm distal from the proximal stump. ^*^Welch’s t test

In contrast, in the histological evaluation (Fig. 6), the mean number of axons was 1036.40 ± 383.61/mm^2^ in group A and 680.44 ± 248.87/mm^2^ in group B at 5 mm distal to the stump (*p* = 0.035) and 1026.13 ± 500.00/mm^2^ in group A and 752.12 ± 343.33/mm^2^ in group B at 10 mm distal to the stump (*p* = 0.2). These findings showed significant differences between the two groups at 5 mm distal to the stump (Fig. 7).

**Fig. 8:**
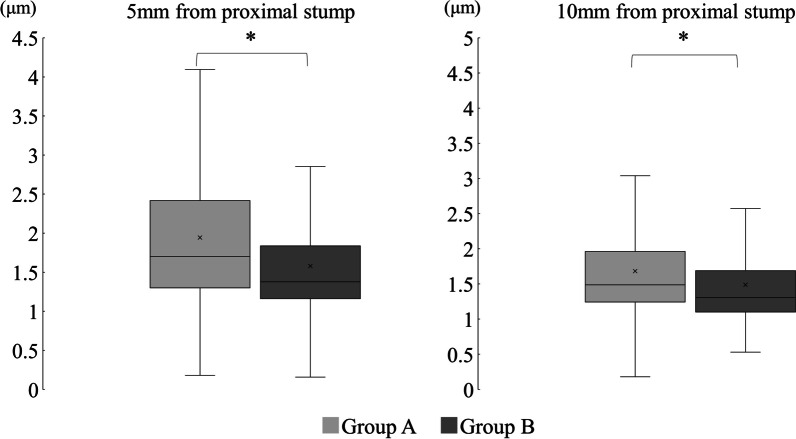
Comparison of the average axonal diameter. There were significant differences between the two groups at 5 mm and 10 mm distal from the proximal stump. ^*^Mann–Whitney U test

The average axonal diameter was 3.38 ± 1.88 μm in group A and 2.64 ± 1.39 μm in group B at 5 mm distal to the stump (*p* < 0.01) and 2.69 ± 1.13 μm in group A and 2.32 ± 1.05 μm in group B at 10 mm distal to the stump (*p* < 0.01). These findings showed significant differences between the two groups in the axonal diameters at both 5 mm and 10 mm distal to the stump (Fig. 8).

## Discussion

In this study, we observed that at 5 mm distal from the stump, the Ochiai suture method resulted in a higher number of axons and a larger diameter compared to the perineural suture method. Similarly, at 10 mm distal from the stump, the axon diameter was larger after the Ochiai suture method than after the perineural suture method. Particularly, since the femoral and sciatic nerves were cut at the same level in all cases, and the distal nerve length was adjusted to suture at the same level in each group, histological evaluations were performed at the same point in both cases. These findings suggest that the Ochiai suture method may lead to better axon regeneration than the perineural suture method for size-mismatched nerve transfers.

While there have been many clinical case series and case–control studies on nerve transfer, most of them have not described the specific suture method used; consequently, we consider that the use of the traditional perineural nerve suture method is most probably employed in those studies. Notably, the nerve suture technique is a crucial factor that influences axon regeneration. Recently, there have been few reports on the use of nerve suture methods [10]. The three most common standard nerve suture techniques usually employed are the epineural, fascicular, and epi-perineural sutures. Among them, the epineural suture is widely used in nerve end-to-end sutures because of its practicality and effectiveness [11]. However, despite the challenges arising from size-mismatched nerve sutures, these standard suture methods are widely used in nerve transfer.

Compared to these standard methods, the Ochiai suture method has several merits. First, the fascicular adaptation sites of smaller donor nerves are centralized at the larger recipient nerves; thus, it is expected that equal nerve regeneration will occur. However, if the standard epineural nerve suture method was performed, unequal nerve regeneration might occur because the donor nerve is inclined toward one area. Second, the adaptation site was covered with an epineural skirt which acted as a nerve conduit to promote nerve regeneration and prevent adhesion to the surrounding tissue. Third, no foreign materials in the fascicular adaptation site have been reported to inhibit nerve regeneration through foreign body reactions [12, 13]. Fourth, there is no tension at the repair site because no suturing was performed at the adaptation site. Besides, research has shown that less tension at the repair site leads to better regeneration [14, 15].

Regardless of the above merits, this study has several limitations. First, immunobiological evaluations were not performed, which restricted our assessment to only axonal number and diameter. Second, femoral-to-sciatic nerve transfer is not usually performed in humans. Third, the lack of functional differences observed during the SFI test and muscle wet-weight determination of the gastrocnemius may be attributed to the femoral nerve's small size or the distance from the suture point to the motor point, which was too long to obtain useful gastrocnemius muscle power.

## Conclusions

This study revealed that the Ochiai suture method for size-mismatched nerves demonstrated superior nerve regeneration than the perineural suture method. This finding confirms that Ochiai suture method can be used for any size-mismatched nerve transfer and may lead to effective motor function restoration for size-mismatched nerve transfer.

## Data Availability

The datasets used and/or analyzed during the current study are available from the corresponding author on reasonable request.

## Abbreviation

SFI: Sciatic functional index
